# The Indirect Impact of Educational Attainment as a Distal Resource for Older Adults on Loneliness, Social Isolation, Psychological Resilience, and Technology Use During the COVID-19 Pandemic: Cross-Sectional Quantitative Study

**DOI:** 10.2196/47729

**Published:** 2023-11-24

**Authors:** Eric Balki, Niall Hayes, Carol Holland

**Affiliations:** 1 Department of Health Research Faculty of Health and Medicine Lancaster University Lancaster United Kingdom; 2 The Directorate Nottingham Trent University Nottingham United Kingdom

**Keywords:** pandemic, educational attainment, isolation, loneliness, socioemotional needs, social engagement, technology use, older adults, psychological resilience

## Abstract

**Background:**

During the COVID-19 pandemic, government-mandated social distancing prevented the spread of the disease but potentially exacerbated social isolation and loneliness for older people, especially those already vulnerable to isolation. Older adults may have been able to draw from their personal resources such as psychological resilience (PR) and technology use (TU) to combat such effects. Educational attainment (EA) or early-life EA may potentially shape later-life personal resources and their impact on the effects of the pandemic lockdown on outcomes such as loneliness. The developmental adaptation model allows for the supposition that social isolation, TU, and PR may be affected by early EA in older adults.

**Objective:**

This study examined the indirect impact of EA on pandemic-linked loneliness in a sample of older adults. The developmental adaptation model was used as the conceptual framework to view EA as a distal influence on loneliness, social isolation, PR, and TU. We hypothesized that EA would predict TU and PR and have a moderating impact on social isolation and loneliness. We also hypothesized that PR and TU would mediate the effect of EA on loneliness.

**Methods:**

This was a cross-sectional observational study, in which data were gathered from 92 older adults aged ≥65 years in the United Kingdom from March 2020 to June 2021, when the country was under various pandemic-linked social mobility restrictions. The data captured demographic information including age, gender, ethnicity, and the highest degree of education achieved. The University of California Los Angeles Loneliness Scale, Connor–Davidson Resilience Scale, Lubben Social Network Index, and Technology Experience Questionnaire were used as standardized measures. Pearson correlation, moderation, and mediation regression analyses were conducted to investigate the hypotheses.

**Results:**

We found a higher prevalence of loneliness in older adults than in prepandemic norms. EA was correlated with greater TU and PR and moderated the impact of social isolation on loneliness. PR mediated and TU partially mediated the relationship between EA and loneliness.

**Conclusions:**

Early-life EA was confirmed as a distal resource for older adults and played an indirect role in affecting loneliness levels during the pandemic. It has an impact on present-day personal resources, such as PR and TU, which affect loneliness and also moderate the impact of social isolation on loneliness. Policymakers should be aware that older adults with low levels of EA may be more vulnerable to the harmful impacts of loneliness when isolated by choice.

## Introduction

### Overview

The COVID-19 pandemic has been a severe health and socioeconomic crisis that created an immense amount of pressure with isolation, contact restrictions, and economic shutdown, transforming the psychosocial environment [1]. Public health responses focused on reducing community spread and increasing physical distance (“social distancing”). This helped reduce exposure to the virus and manage pressure on health care facilities. These measures included stay-at-home orders, cancellation of social activities and events, and limited to no visiting permitted.

The older adult population was disproportionately impacted by the pandemic. Older adults were particularly vulnerable to contracting COVID-19 and it progressing to a life-threatening state [2]. Age has also been strongly associated with the risk of fatality due to contracting COVID-19 [3] (eg, UK patients aged >80 years were found to be 20 times more likely to die from COVID-19 than those aged 50-59 years). Although social distancing measures during the pandemic may have been highly advantageous in reducing the spread of the disease, they did lead to an objective lack of interaction, particularly in older adults [4]. Being restricted physically in one location amplified negative outcomes for older adults and led to increased loneliness [5].

Before the pandemic, social isolation and its link to loneliness were well known as a severe public health concern for older adults with links to serious physical and mental health comorbidities [6-9]. Although social isolation may not be emotionally distressing for all, it is a known health risk factor comparable with smoking [10] and is linked to limited access to caregivers; the lack of financial, clinical, or emotional support; and exacerbating loneliness in older adults. Shelter-in-place and social distancing orders may have magnified the problem further, with older adults being restricted to their homes and not being able to see family and friends in person during the pandemic.

Several studies have examined loneliness and social isolation during the pandemic; however, this body of literature is still in its infancy, especially in relation to coping strategies among older adults [11,12]. Loneliness, already at high levels among the older adult population globally [13], is multifaceted and complex. Most studies examining loneliness during the pandemic have reported that loneliness levels in older adults were negatively affected by the COVID-19 pandemic compared with prepandemic norms [14-16]. Some causes identified for increased loneliness emanated from the inability to cope emotionally, social isolation resulting from stay-at-home orders or social distancing, insufficient social support, and inadequate access to communication technologies. Most older adults usually rely on a small number of informal support sources (eg, acquaintances, friends, and caregivers) to overcome hardship or adversity [17]. Limited exchanges with social network members may increase perceptions of stress, social isolation, and loneliness. Recent studies suggest that personal resources, such as technology use (TU) [18] and psychological resilience (PR) [19], may play a pivotal role in protecting older adults from isolation and loneliness. However, currently, there is very limited evidence on how educational attainment (EA) as a distal resource could have impacted these personal resources and whether past EA had an indirect impact on loneliness during the pandemic.

Successful aging is defined as high social, physical, and psychological functioning in old age [20]. The focus of the concept of successful aging is the expansion of healthy and functional years in the life span [21]. Previous studies have found that from a developmental perspective, successful aging results from satisfaction with increasing age-related demands for cultural input, where culture refers to the entirety of social, material, psychological, and symbolic (knowledge-based) resources and is known to reduce psychological vulnerability [22]. This posits the notion that individuals draw from and conserve personal (physical, material, social, and psychological) resources acquired during their life span and use them more as they age, especially in times of difficulty and stress to maintain or restore successful aging.

TU is a resource that could have mitigated the impact of the pandemic on loneliness, and social isolation is TU [11,12]. Previous studies have consistently highlighted the potential positive impact of TU on well-being, with a reduced probability of social isolation due to an increase in connections and a decrease in loneliness [23-26]. Videoconferencing apps (such as Microsoft Teams), instant messaging apps (such as WhatsApp), and services (such as Zoom) grew in popularity during the COVID-19 pandemic, for both social and business use [27]. Digital communication technology (DCT) can also increase social support from family, friends, social groups, and communities, which can be important when someone meets the criteria of being disconnected [28-31]. Recent studies that have examined TU in older adults during the pandemic have largely reported similar findings, for example, Yang et al [32] found that higher TU predicted lower levels of loneliness. Llorente-Barroso et al [33] reported that TU enabled closeness to family and friends. The use of such technology might have mitigated the impact of social isolation with it being used in place of previous in-person meetings among friends, family, and colleagues [29-31,34]. Group-based web-based meetings were being orchestrated through videoconferencing programs, as well as religious gatherings, yoga [18], or playing web-based games [35], all of which may have decreased feelings of loneliness.

Another resource that could have mitigated the impact of the pandemic on loneliness and social isolation is PR [36]. Psychologists have defined resilience as the ability to adapt well in the face of difficulties, tragedy, trauma, threats, or a significant source of stress [37] or as a resource that helps adapt successfully to any disturbances and shelter from significant challenges that threaten sustainability, stability, and growth [38]. PR can also be considered as either a process, outcome, or a trait acquired during one’s lifetime [38]. As a process, it has been referred to as a dynamic process encompassing positive adaptation when facing significant adversity [39]. Resilience can be thought of as “bouncing back” from such difficult circumstances, and it can also involve personal growth and reflection [40]. Other studies that have demonstrated an increase in loneliness in older adults over time have consistently reported the need to improve the understanding of how PR could be an influencing factor, and there is good evidence that it can protect and even mediate relationships between health-related issues and loneliness [41-45]. Strong social ties have also been described as a key feature of PR [46], which may be improved through interventions [47]. Therefore, for older adults, PR represents an ability to return to equilibrium when they find themselves in difficult circumstances or situations [48-55]. Therefore, PR could have acted as a defense mechanism against increased loneliness and social isolation among older adults during the pandemic.

Factors that may enhance PR include concern for generativity (a concern in establishing and guiding the next generation), frequent religious attendance, and an ability to develop meaning in life when facing adversity [55]. Resilience could also be gained from one’s social network and support, and perhaps the ability to remain connected with network members, potentially through technology, may have impacted resilience levels [56], indicating that those with higher PR may have used technology more.

However, despite EA being assumed as a resource acquired in the past for older adults, there is an expectation that this investment in knowledge and skills has long-term beneficial returns [57]. Dannefer [58] envisaged EA to be a mechanism of “cumulative advantage” across the life course, describing it as a process of “accentuation,” that is, a process of accumulation throughout the life course. This hints at a potential pathway for early-life experiences shaping later psychosocial states including loneliness, in ways that also influence the acquisition of and the ability to use personal resources later in life. Studies have acknowledged that the association between distal experiences and better outcomes in older populations is mediated by psychosocial variables such as PR [59]. EA may also have an indirect effect on developmental outcomes later in life, including social isolation, how much older adults use technology, and how they draw upon PR to mitigate the circumstances of the pandemic. Therefore, in this study, we were particularly interested in EA as a distal resource in the past, which could have had a continued impact on present-day loneliness mediated by proximal personal resources in older adults.

EA is considered cognitively, socially, and physically stimulating, and has been selected as one of the most representative factors of cognitive reserve, which describes a person’s PR and ability to withstand and adapt [60]. Mirowsky and Ross [61] argued that EA influences adults in ways that are diverse, omnipresent in adult life, cumulative, and positive. It creates desirable outcomes because it encourages individuals to evaluate, acquire, and use information and personal resources in effective ways to tap the power of knowledge and help develop the learned effectiveness that enables goal-orientated self-direction amplifying the impact of personal resources [62]. In a study on unmarried older adults, Bishop and Martin [63] found that EA had a negative indirect effect on loneliness (causing it to increase) through neuroticism. Other studies have provided evidence of a link between childhood EA and the development of emotional skills necessary to seek and maintain high-quality social relationships, both in childhood and adult life [64,65]. Kung et al [66], expanding on this, confirmed EA was strongly linked to a lower level of loneliness.

If EA as a distal resource is found to have a significant relationship with proximal personal resources impacting loneliness in older adults while also moderating the impact of social isolation, it may be considered a useful tool to identify older adults who may be more vulnerable to the negative impact of loneliness and isolation. Although there have been studies that have examined loneliness and social isolation in older adults during the pandemic, to the best of our knowledge, this is the first study to examine the impact of EA as a distal resource on levels of social isolation and loneliness during the pandemic, along with its impact on proximal resources such as TU and PR that could help mitigate their effect.

Although an explicit link between EA and PR in the literature is scarce, some studies have strongly hinted at its presence. For example, lower academic dropout rates have been associated with resilience [67], and there has been a consistent relationship between academic success and resilience [68,69]. Backmann et al [70] identified the link between positive outcomes in EA and PR through a link between positive personality traits such as emotional stability, openness, and conscientiousness being related to PR, and observed that EA and PR may modify each other’s effects, hinting at a potential mediation pathway with other measures. An additional link connecting EA and PR and their impact on loneliness stems from studies that have found a link between EA and higher cognitive function later in life, which is necessary for PR [71].

EA could also be a bridge that enables older adults to navigate TU. Technology represents advances in skills, knowledge, and abilities that change the manner in which a person performs tasks. It can be considered an acquired personal resource that can make certain tasks easier, more efficient, safer, and perhaps pleasurable. Kämpfen and Maurer [72] in assessing early education of older adults on computer technology adoption and internet use found that 1 additional year of education resulted in almost 9% increase in the probability of having ever used the internet and in an increase of 12% in the probability of having at least “good” computer skills. Other studies noted that the quality of TU was also impacted by EA, although the causes have not been explored in detail [73-75]. This highlights the importance of EA as a distal resource for later-life TU and adoption, and the importance of bridging the knowledge gap about it and its influence on proximal factors.

### Conceptual Framework

Studies that have examined stressful circumstances have discovered that personal resources (eg, resilience, self-efficacy, and perceived control) and social resources (eg, emotional support and social connectedness) can buffer against the negative impacts of stressful situations [76]. Older adults are generally a group of individuals that are under the increasing threat of actual or potential loss of resources over their life span (based on 5 dimensions including age-associated decline and loss of health resources, early cumulative factors, reduction and loss of economic resources linked to changes in job or retirement, loss of social support resources due to complexities revolving around access, and the personal experiences of losing friends and family members). Theories of aging such as the life course theory proposed by Elder [77] also reflect the age-linked loss of resources and perceived old age, emphasizing that one accumulates resources throughout one’s life course, but there is a difference between people in how much they possess and use them depending upon the accessibility and availability of resources, resulting in differing levels [78]. When under threat or in a stressful environment such as the pandemic, individuals would feel encouraged to retain, protect, and foster valued resources for anticipated future requirements [57]. When investment in the acquisition of such resources fails in early life, vulnerability to stressors can lead to negative psychological outcomes such as loneliness [79]. Therefore, it can be argued that past life experiences and investment in resources can reduce vulnerability later in life.

Therefore, EA may be linked to the positive evaluation of contextually stressful or difficult circumstances and experiences and then adaptation to be able to deal with those circumstances. Adaptation is defined as a psychosocial adjustment to changing situations or circumstances [80]. The developmental adaptation model (DAM) by Martin and Martin [81] proposed a mechanism for how early-life experiences can be linked to current circumstances and the ability to adapt and cope with them. Their model synthesized a mechanism by which early experiences or distal influences could impact current outcomes through their action on proximal resources [82-84]. The DAM, with its pathways, can serve as the basis for the exploration of developmental trajectories based on life histories as well as present resources that play a crucial role in successful adaptation.

The model combines distal development influences with proximal influences, behavioral coping mediators, and developmental outcomes. The 2 central components are stress and ability to cope and life span and life course time frame. The model provides for the argument that distal resources influence long-term outcomes, such as loneliness, through action on proximal resources. It allows us to hypothesize the role that could be played by education on resilience and developmental outcomes (ie, loneliness) linking it to higher cognitive function later in life, which is necessary for increased resilience and TU. Essentially, the effect of distal, personal, social, and external resources may lead to an impact on later-life outcomes.

Incorporating DAM in this research allowed us to uncover indirect pathways of EA’s impact on loneliness and mediating pathways proximal (social isolation, PR, and TU) variables relative to the current outcomes on loneliness during the COVID-19 pandemic.

### Aims

We posit that early EA is a distal resource that affects present-day loneliness. PR and TU, both as personal resources that older adults potentially possess, surface as factors that could directly impact loneliness and potentially mediate the relationship between EA and loneliness. This study was conducted to answer the following research question: “How did past EA as an indirect distal resource influence loneliness through its relationship with social isolation, PR, and TU among older adults during the COVID-19 pandemic?”

To bridge this gap in the literature, we aimed to explore the findings and limits of current knowledge on the indirect impact of EA as a distal resource on loneliness in older adults. We expected EA to influence PR and loneliness, as well as PR and TU, to play a mediating role between EA and loneliness. Furthermore, we expected EA to play a moderating role between social isolation and loneliness. The direct impacts of PR and TU on loneliness were also explored.

We set out to test the following hypotheses:

1. Higher EA is correlated with higher PR and lower loneliness.
2. Higher PR predicts greater TU.
3. EA will predict greater TU after controlling for PR.
4. EA will moderate the impact of social isolation on loneliness.
5. TU will mediate the relationship between EA and loneliness.
6. PR will mediate the relationship between EA and loneliness.

## Methods

### Setting

This was a quantitative cross-sectional observational study. The STROBE (Strengthening the Reporting of Observational Studies in Epidemiology) checklist was used [85].

### Participants

Participants were recruited from England, United Kingdom, with a large majority (n=92, >80%) located in the northwest. The study was conducted during the mandated social distancing period of COVID-19 (March 16, 2020-June 21, 2021), with social mobility largely restricted.

Participants were required to be living in their own homes, be aged ≥65 years (age inclusion criterion specified by the American Psychological Association and APA, 2002) [85], and proficient in English. Participants living in nursing homes, living in care homes, or with a history of mental health issues were not considered eligible for this study.

Recruitment was conducted by advertising through resource centers for older adults, housing associations, third-sector organizations, social activity clubs, and local older adult groups via a personal approach and word-of-mouth recommendations from participants. Participants would either call and leave a voicemail or send an email if they wanted to participate. A call back would be made to determine eligibility, where participants were asked to complete the required informed consent process.

To determine the minimum size of the research sample necessary for the empirical verification of the tested moderation model, G*Power software was used with an effect size of f_2_=0.15, power=0.80, and 3 predictors, using the multiple regression design option for analyses. This resulted in a sample size of 87. A total of 110 participants were recruited for the study. However, 18 participants did not complete all the questionnaires and were excluded. The sample included 92 people aged between 65 and 92 (mean 74.6, SD 7.23) years. All participants were identified as either male or female, with more women (55/92, 60%) than men. More than 89% of the participants were White, with less than 11% from minority ethnicities (n=7 British Asian; n=3, British Black). In North West England, ≤1.4% of the older adult population is British Black, and less than 6.2% is British Asian [19] therefore, our sample seemed to be representative of areas from which participants were recruited. Having collated various demographic information such as age, gender, ethnicity, and education level, we were able to ascertain that the participants emanated from diverse sociodemographic backgrounds.

### Variables and Measures

Participants completed a background health questionnaire that was developed based on the *SAGE Encyclopedia of Communication Research Methods* [86] and included capturing age, gender, ethnicity, sexual orientation, and EA.

Loneliness was measured using the 20-item University of California Los Angeles Loneliness Scale [87] with scores ranging from 20 to 80. Higher scores reflect higher loneliness (Cronbach α=.88).

PR was assessed using the 10-item Connor–Davidson Resilience Scale (CD-RISC) [88], in which items were rated on a 5-item scale ranging from 1 (strongly disagree) to 5 (strongly agree). The possible scores ranged from 0 to 60, with higher scores reflecting greater resilience (Cronbach α=.88).

The 12-item Lubben Social Network Index [89] was used to measure social network size and support, reporting on social isolation levels. The possible score range was 0 to 60, with a higher score indicating more social engagement and greater social connectedness (Cronbach α=.88).

TU was measured using the Technology Experience Questionnaire [90], in which participants were presented with a list of technologies (representing communication technology, computer technology, everyday technology, health technology, recreational technology, and transportation technology) and asked to indicate their familiarity with each on a 5-point scale. Scores ranged from 0 to 180, with higher scores indicating greater use of and familiarity with technology (Cronbach α=.84).

EA was defined as the highest level of academic achievement experienced by participants, and options included no formal education, high school equivalent (GCSEs and O-Levels), college or undergraduate degree, postgraduate degree, and advanced postgraduate degree (doctorate or equivalent).

### Ethical Considerations

Participants received an information sheet before providing consent either via email or on the phone and were allowed to ask any questions. They were informed of their right to withdraw at any point in the research and given advice about anonymity. Ethical procedures aligned with the British Psychological Society guidelines and the study received ethical approval from the University Faculty Research Ethics Committee (FHMREC19121). Data were captured over the phone after the identity of the participant was confirmed and were recorded in spreadsheets and anonymized.

### Procedure

The surveys were conducted over the telephone, gathering information on loneliness, social isolation, TU, and PR in addition to basic demographic information (age, education, and sex). Google Analytics was used to record and tabulate the data, with further analysis performed using SPSS (version 28; IBM Corp). Participants completed the assessments over 14 months, spanning various levels of COVID-19 pandemic–related lockdown measures.

### Statistical Methods

All analyses were performed with a 95% probability and a minimum significance level of *P*<.05. There were no missing data identified among the observations. The variables EA, loneliness, TU, social isolation, and PR were screened for skewness and kurtosis to assess the deviation of their distributions from normality using a histogram with simulated overlapping normal curves. The homoscedasticity of the residuals was checked using standardized residual versus standardized predicted plots. Using the Mahalanobis distance (*P*<.001) and the Cook distance, we checked whether there was a linear relationship between the dependent variable and each of the independent variables using a scatterplot matrix of dependent and continuous independent variables to see if there were any significant outliers, high leverage points, or highly influential points. None of the patients were considered necessary for removal. Confirmation of the independence of observations and the assumption of no autocorrelation in residuals were checked using the Durbin-Watson d statistic.

The initial descriptive analyses included frequencies, means, and SDs. Pearson product-moment correlation coefficients were calculated to determine if there was an association between the dependent and continuous variables, which could further explain the relationships found in the proposed hypotheses. The same correlational analysis was used to determine whether EA was correlated with PR (hypothesis 1).

Simple linear regression models were built to evaluate whether PR predicts a greater TU to examine hypothesis 2. Whether EA predicts TU after controlling for PR (hypothesis 3) was assessed using hierarchical multiple linear regression analysis. TU was set as the dependent variable, EA was set as the independent variable, and PR was set as the control variable. The associated predictor variables were entered into the model, and a backward elimination approach was used, removing any variable with Cronbach α>.15.

The PROCESS macro for SPSS (version 3.2) by Hayes [91] essentially computes regression analyses containing various combinations of mediators, moderators, and covariates. With model 1, it was applied to investigate the moderating effects of EA on the relationship between social isolation and loneliness as per hypothesis 4. If the standardized coefficients of the interaction terms were significant (*P*<.05) or marginally significant (*P*<.09), we conducted a simple slope test to examine the interaction effect and further explain the moderating effect.

To test whether TU mediates the relationship between EA and loneliness (hypothesis 5) and whether PR mediates the relationship between EA and loneliness (hypothesis 6), we used the PROCESS macro model 4 by Hayes [91], which allows us to test the mediating relationship with bootstrap CIs for an indirect effect. We applied a bootstrapping approach to determine the indirect effect for each of the bootstrapped 5000 sample items from the original data set using stochastic sampling with replacement. As a nonparametric resampling procedure, bootstrapping is considered the most powerful method for small samples because it is the least vulnerable to type 1 errors. If the CIs did not include 0, the effects were considered significant (*P*<.05).

## Results

### Overview

The calculated means and SDs and the maximum and minimum as basic descriptive statistics are shown in Table 1.

Participants demonstrated moderate to high levels of loneliness with 44% of older adults demonstrating loneliness scores above 50 [87]. The Social Network Scale indicated by Lubben [89] that most participants reported good levels of social connectedness, with 82% scoring above 30 points. For PR, most participants (≥57%) scored above 25, demonstrating high levels of resilience [34]. Regarding TU, most participants scored ≥125 (n=92, 56%), demonstrating high use and familiarity with technology in general [90]. However, we noted a binormal distribution with a statistically significant number of participants at the end of the spectrum of low technology scores (n=92, 32%) lower than 120, which indicated low familiarity and use of technology [90]; 64% (n=92) of participants scored ≥2 as far as EA, indicating the completion of A-Levels or a high school equivalent, and almost 38% (n=92) of the participants had an undergraduate degree or higher.

Pearson correlation coefficients were calculated to examine the relationship among EA, loneliness, technology, PR, and social isolation. The results of the correlational matrix analysis are presented in Table 2.

**Table 1:** Descriptive statistics (n=92).

Scores	Minimum	Maximum	Values, mean (SD)
UCLA^a^ Loneliness Score	20	80	47.49 (17.814)
Lubben Social Network Scale	1	49	26.91 (15.304)
Psychological resilience	5	36	21.76 (10.543)
Technology use	48	175	116.87 (40.951)
Educational attainment	0	4	N/A^b^

^a^UCLA: University of California Los Angeles.

^b^N/A: not applicable.

**Table 2:** Correlational analysis between variables (N=92).

	UCLA^a^ Loneliness Score, *r*	Lubben Social Network, *r*	Psychological resilience, *r*	Technology experience, *r*	Educational attainment, *r*
UCLA Loneliness Score	1	−0.853^b^	−0.885^b^	−0.631^b^	−0.674^b^
Lubben Social Network	−0.853^b^	1	0.866^b^	0.557^b^	0.689^b^
Psychological resilience	−0.885^b^	0.866^b^	1	0.610^b^	0.773^b^
Technology use	−0.631^b^	0.557^b^	0.610^b^	1	0.588^b^
Educational attainment	−0.674^b^	0.689^b^	0.773^b^	0.588^b^	1

^a^UCLA: University of California Los Angeles.

^b^*P*<.01 (2-tailed).

### Hypothesis 1: EA Is Correlated With Higher Levels of PR and Lower Loneliness

As shown in Table 2, EA and PR were statistically significantly and positively correlated (*r*=0.773; *P*=.007). EA and loneliness were statistically significantly and negatively correlated (*r*=−0.674; *P*=.006). This meant that higher EA correlated with higher PR scores and lower loneliness, thus supporting our first hypothesis.

### Hypothesis 2: PR Will Predict Greater TU

The results examining whether PR predicted TU (hypothesis 2) were checked using simple linear regression analysis. TU was set as the dependent variable, and PR was set as the independent variable. Table 3 presents the coefficient results of the simple linear regression analysis.

The results showed that PR (B=2.368, *t*_90_=7.298, *P*<.001) was a significant predictor of TU. The results of the ANOVA test for the significance of the regression model showed that the whole model was significant (*F*_1,90_=53.261; *P*<.001). PR accounted for almost 37.2% of the variance (*R*^2^=0.372) of TU. Therefore, we can conclude that PR significantly predicted TU.

**Table 3:** Model output and coefficients of simple linear regression model on technology use.

Regression equation (dependent and independent variable)			Overall fit											Significance of regression coefficient		
			*R*		*R* ^2^		*F* test		*df1*		*df2*		Β			*t* test (*df*)
**Technology experience**			0.61		0.37		53.26		1		90		N/A^a^			N/A
	Intercept											65.33			8.33^b^ (90)	
	Psychological resilience											2.37			7.30^b^ (90)	

^a^N/A: not applicable.

^b^*P*<.001.

### Hypothesis 3: EA Will Predict TU After Controlling for the Impact of PR

The results examining whether EA predicts TU use after controlling for PR (hypothesis 3) were checked using hierarchical multiple linear regression analysis. TU was set as the dependent variable, EA was set as the independent variable, and PR was set as the control variable. Table 4 shows the coefficient results of the multiple regression analysis.

The results showed that both PR (B=1.501, *t*_90_=3.001, *P*=.008) and EA (B=9.348, *t*_90_=2.243, *P*=.04) were significant predictors of TU. The results of the ANOVA for the significance of the regression models showed that the combined effect of both predictors was significant (*F*_2,89_=30.339; *P*<.001). EA alone in the model accounted for almost 34.5% of the variance (*R*^2^=0.345). Adding PR to the multiple regression model changes the value of *R*^2^ by 0.06 (*P*=.04). Therefore, we can say that after PR is controlled for and EA scores significantly predicted TU, thus confirming our third hypothesis.

**Table 4:** Model output and coefficients of multiple linear regression model on technology experience.

Model and regression equation (dependent and independent variable)		Overall fit						Significance of regression coefficient	
		*R*	*R* ^2^	*F* test	*df1*	*df2*	Β		*t* test (*df*)
**Technology use**		0.59	0.35	47.45	1	90	N/A^a^		N/A
	Intercept					90	83.80		14.14^b^ (90)
	Educational attainment					90	19.01		6.89^b^ (90)
**Technology use**		0.64	0.40	30.34	2	89	N/A		N/A
	Intercept					89	67.94		8.76^b^ (90)
	Psychological resilience					89	1.50		3.00^c^ (90)
	Educational attainment					89	9.35		2.24^d^ (90)

^a^N/A: not applicable.

^b^*P*<.001

^c^*P*<.01.

^d^*P*<.05.

### Hypothesis 4: EA Will Moderate the Impact of Social Isolation on Loneliness

**Figure 1:**
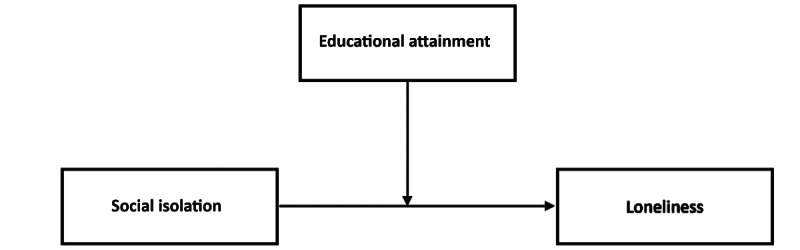
The moderating role of educational attainment on social isolation.

Model 1 was used in the PROCESS 4.0 macro to examine the moderation effect of EA on social isolation for loneliness, as proposed in hypothesis 3 [92] and as shown in Figure 1.

All continuous variables were converted into *z* scores for use in the model; *z* scores describe the position of raw scores in terms of their distance from the mean when measured in SD units and standardize the distribution. As shown in Table 4, the unconditional interaction of EA and social isolation was not significant (β=.14, *t*_90_=1.7926, *P*=.07), and EA did not seem to have a moderating effect on the impact of social isolation on loneliness.

**Figure 2:**
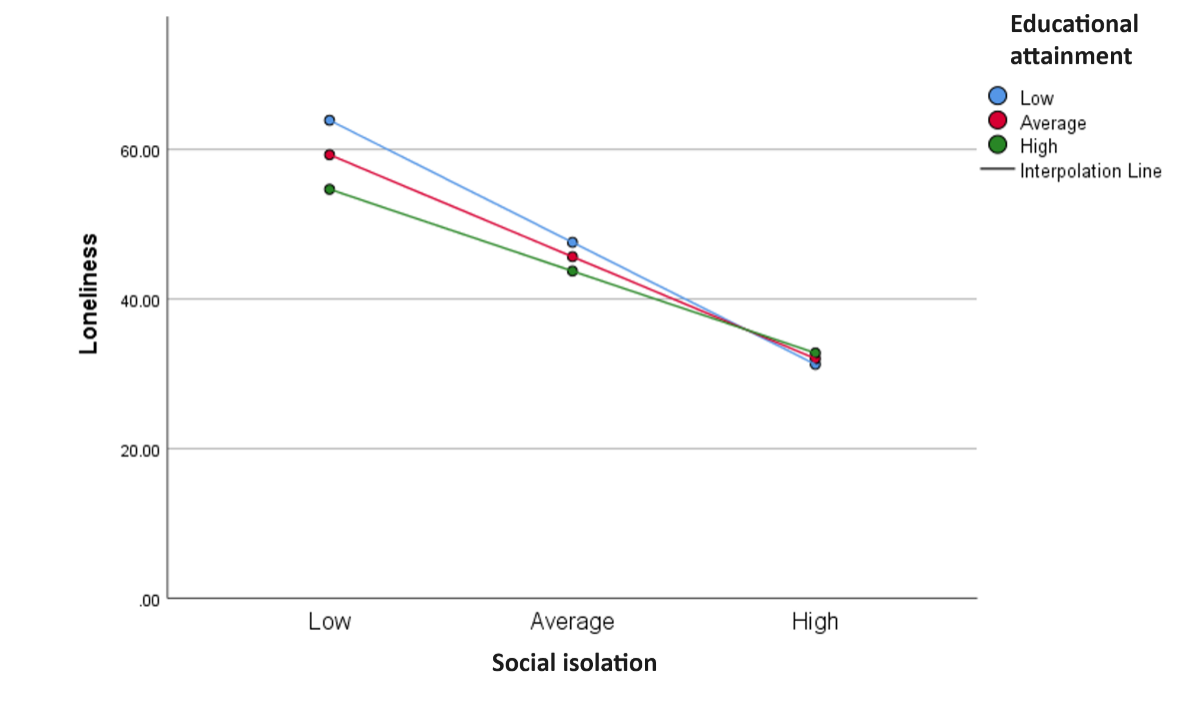
The moderating effect of educational attainment on social isolation. The graph shows the grouped scatter of average loneliness score by social isolation by education level.

To check whether there was any conditional interaction between EA and social isolation, we used the simple slope test to further analyze the moderation impact as can be seen in Figure 2.

When EA was high, social isolation and loneliness were significantly negatively correlated (βsimple(M+1SD)=−0.72, *t*_90_=−6.05, *P*<.001). However, when EA was low, the correlation between social isolation and loneliness was not significant (βsimple(M-1SD)=−1.07, *t*_90_=−1.88, *P*=.06). Thus, we can conclude that EA moderates the impact of social isolation on loneliness when EA is high but does not have an impact when EA is low. This confirms our third hypothesis and offers an explanation for why a moderation impact was not observed initially in the unconditional analysis, as only higher EA seems to have a moderating impact, and at lower levels, the impact is not discernible.

### Hypothesis 5: TU Will Mediate the Relationship Between EA and Loneliness

**Figure 3:**
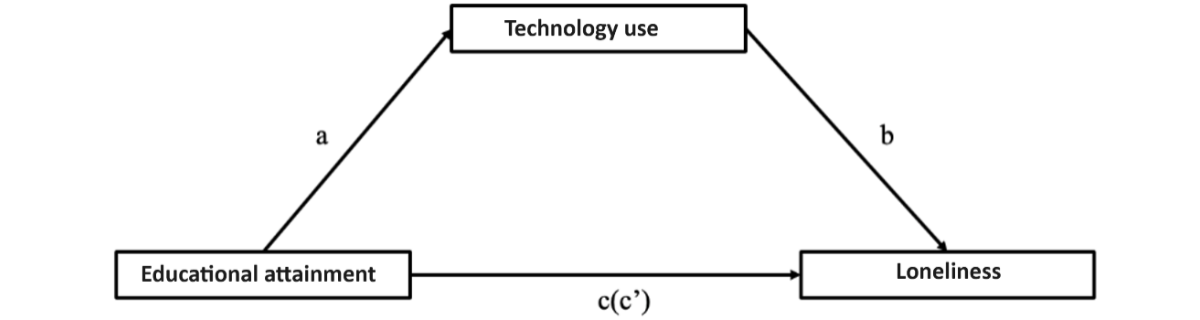
The mediating role of technology use between educational attainment and loneliness. Here, path a represents the effect of educational attainment on technology use, path b represents the effect of technology use on loneliness, and path c represents the total effect of educational attainment on loneliness. Path c’ represents the direct effect of educational attainment on loneliness.

Using model 4 in the PROCESS macro 4.0 compiled by Hayes [93], we tested the mediating effect of TU between EA and loneliness, as shown in Figure 3, and the results are summarized in Table 5.

EA had a significant predictive effect on loneliness (path c; B=−0.67, *t*_90_=−8.6616, *P*<.001), and when TU (the intermediary variable) was inserted, the direct predictive effect of EA on loneliness (Path c′) was still significant (B=−0.46, *t*_90_=−5.2103, *P*<.001). The positive predictive effects of EA on TU (path a; (B=0.59, *t*_90_=6.8884, *P*<.001) were significant, and the negative predictive effects of TU on loneliness were also significant (path b; B=−0.3588, *t*_90_=−4.033, *P*<.001; Table 6).

In addition, the direct effect of EA on loneliness was established (upper and lower limits of bootstrap at the 95% CI −9.01 to −4.04 did not contain 0), and the mediating effect of TU was also significant (upper and lower limits of bootstrap at the 95% CI −5.67 to −0.66 did not contain 0); the bootstrapped mediation indicates the presence of partial mediation of TU on the relationship between EA and loneliness (Table 7).

This intermediary effect accounted for 31.26% of the total effect. Therefore, we conclude that our fifth hypothesis is partially supported.

**Table 5:** Moderation analysis for the effect of social isolation on loneliness with educational attainment as a moderator.

		Overall fit						Significance of standardized coefficient	
		*R*	*R* ^*2*^	*F*	*df1*	*df2*	B		*t* test (*df*)
**Independent variable**		0.87	0.75	88.7417	3	88	N/A^a^		N/A
	Social isolation (ZSI)						−.89		−10.26^b^ (88)
	Education (ZEA)						−1.52		−1.35 (88)
	ZSI*ZEA						0.14		1.79 (88)

^a^N/A: not applicable.

^b^*P*<.001. All variables in the model were standardized and entered into the regression equation.

**Table 6:** Intermediary model test of technology use^a^.

Regression equation (dependent variable and independent variable)			Overall fit											Significance of regression coefficient		
			*R*		*R* ^*2*^		*F* test		*df1*		*df2*		Β			*t* test (*df*)
**Loneliness**			0.67		0.45		75.02		1		90		N/A^b^			N/A
	Educational attainment (path c)											−0.67			−8.66^c^ (90)	
**Technology use**			0.59		0.35		47.45		1		90		N/A			N/A
	Educational attainment (path a)											0.59			6.89^c^ (90)	
**Loneliness**			0.73		0.54		52.00		2		89		N/A			N/A
	Technology use (path b)											−0.36			−4.03^c^ (89)	
	Educational attainment (path c’)											−0.46			−5.21^c^ (89)	

^a^All variables in the model are standardized and introduced into the regression equation.

^b^N/A: not applicable.

^c^*P*<.001.

**Table 7:** Decomposition table of the total effect, direct effect, and indirect effect.

	Effect	Boot SE	Boot LLCI^a^	Boot ULCI^b^	Relative effect value (%)
Total effect	−9.4913	1.1	−11.67	−7.31	N/A^c^
Direct effect	−6.5240	1.25	−9.01	−4.04	68.74
Indirect effect	−2.9673	1.27	−5.67	−0.66	31.26

^a^LLCI: lower limit CI.

^b^ULCI: upper limit CI.

^c^N/A: not applicable.

### Hypothesis 6: PR Will Mediate the Relationship Between EA and Loneliness

**Figure 4:**
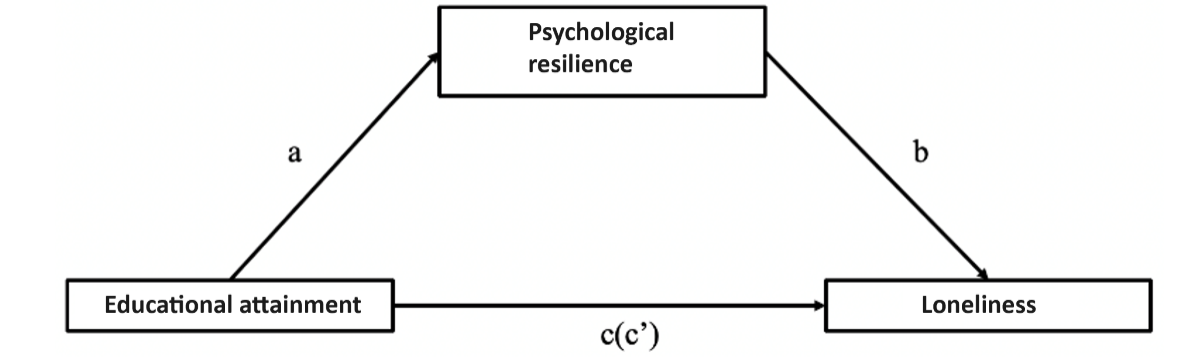
The mediating role of psychological resilience between educational attainment and loneliness. Here, path a represents the effect of educational attainment on psychological resilience, path b represents the effect of psychological resilience on loneliness, and path c represents the total effect of educational attainment on loneliness. Path c’ represents the direct effect of educational attainment on loneliness.

Using model 4 in PROCESS macro40 [93], we tested the mediating effect of PR between EA and loneliness, as seen in Figure 4; the results are summarized in Table 8.

EA had a significant predictive effect on loneliness (path c; B=−0.67, *t*_90_=−8.6616, *P*<.001), and when PR (the intermediary variable) was included, the direct predictive effect of EA on loneliness (path c′) was not significant (B=0.02, *t*_90_=0.3065, *P*=.06), indicating mediation. The positive predictive effects of EA on PR (path a; B=0.77, *t*_90_=11.5513, *P*<.001) were significant, and the negative predictive effects of PR on loneliness were significant (path b; B=−0.9033, *t*_90_=−11.6193, *P*<.001).

In addition, as shown in Table 9, the direct effect of EA on loneliness was not significant (upper and lower limits of bootstrap at the 95% CI −1.84 to 2.51 contained 0), while the mediating effect of PR was significant (upper and lower limits of bootstrap at the 95% CI −12.30 to −7.70 did not contain 0), the bootstrapped mediation indicates the presence of complete mediation of PR on the relationship between EA and loneliness.

This intermediary effect accounted for 96.47% of the total effect. Therefore, we conclude that our sixth hypothesis is supported.

**Table 8:** Intermediary model test of psychological resilience^a^.

Regression equation (dependent variable and independent variable)			Overall fit											Significance of regression coefficient		
			*R*		*R* ^*2*^		*F* test		*df1*		*df2*		Β			*t* test (*df*)
**Loneliness**			0.67		0.45		75.0230		1		90		N/A^b^			N/A
	Educational attainment (path c)											−0.6743			−8.6616^c^ (90)	
**Psychological resilience**			0.77		0.60		133.4333		1		90		N/A			N/A
	Educational attainment (path a)											0.7728			11.5513^c^ (90)	
**Loneliness**			0.89		0.78		160.8685		2		89		N/A			N/A
	Psychological resilience (path b)											−0.9033			−11.6193^c^ (90)	
	Educational attainment (path c’)											0.0238			0.3065 (90)	

^a^All variables in the model are standardized and introduced into the regression equation.

^b^N/A: not applicable.

^c^*P*<.001.

**Table 9:** Decomposition table of the total effect, direct effect, and indirect effect.

	Effect	Boot SE	Boot LLCI^a^	Boot ULCI^b^	Relative effect value (%)
Total effect	9.4913	1.1	−11.67	−7.31	N/A^c^
Direct effect	0.3354	1.09	−1.84	2.51	3.53
Indirect effect	9.8268	1.16	−12.30	−7.70	96.47

^a^LLCI: lower limit CI.

^b^ULCI: upper limit CI.

^c^N/A: not applicable.

## Discussion

### Principal Findings

This study set out to examine the indirect distal impact of EA on loneliness and whether its impact on loneliness is mediated by proximal personal resource predictors including TU and PR. We also examined whether EA moderated the effect of social isolation on loneliness. We used the DAM [81] as our conceptual model, which would allow us to explain how early experiences or distal influences could impact current outcomes through their action on proximal resources.

Our first observation was that we found higher levels of loneliness in this study than in other similar cross-sectional studies. In a recent analysis based on a review of the literature by Hawkley et al [94], the authors found prepandemic loneliness prevalence to be around 25% in older adults, compared with 44% in this study, raising an awareness that loneliness prevalence has been higher during the pandemic. The ability to use technology successfully to adapt to challenging experiences during the lockdown and to maintain social connectedness emerged unsurprisingly as a potential factor that reduced loneliness resulting from social distancing, as TU was correlated with lower loneliness. This was in line with our findings from the literature that highlighted the positive impact of TU on loneliness [23-25,31,32]. TU during the pandemic may have increased the ability of older adults to express themselves efficiently and easily, which has been linked to lower levels of perceived discomfort caused by a difficult situation, which is especially important in times of stress [26]. DCT may have also increased social support from family, friends, social groups, and the community, which can be important when someone meets the criteria of being disconnected [28].

In our first hypothesis, we examined the relationships among EA, PR, and loneliness. EA was found to be positively correlated to PR, that is, that higher attainment was linked to higher PR, and negatively to loneliness, that is, higher attainment was correlated with lower loneliness. Despite the scarcity of the literature exploring the connection between EA and PR, several explanations surfaced when applying the DAM. For example, positive personality traits such as emotional stability, openness, and conscientiousness have been found in people with both high EA and PR [70], showing that people with such traits may be more resilient to adverse circumstances and have good adaptive capabilities. Furthermore, EA may support the development of PR, and PR may help with better outcomes in EA [68,69]. In addition, EA is linked to higher cognitive function later in life, which is necessary for PR and may also impact loneliness [71]. The pandemic was a time when our participants may have been surrounded by a lot of negative stimuli, and therefore EA’s indirect effect on loneliness through neuroticism may also have come into play, as higher EA and higher neuroticism lead to loneliness and the absence of neuroticism can predict lower loneliness [63]. To combat negative stimuli, older adults may have used emotional skills associated with EA and PR to maintain high-quality social relationships during the pandemic [50]. The ability to remain connected with friends and family through technology, sharing positive experiences, social solidarity, the ability to seek support, and generally having a positive mindset as well as applying previously learned skills may all have attributed to EA links with lower loneliness and higher PR [51-53].

Our second hypothesis confirmed the correlational link between PR and TU. Being able to maintain strong social ties has been known to be a key element in people with higher PR [46]. DCTs offer older adults the ability to maintain social connections; thus, older adults with higher PR may have been seeking out the use of technology more [47]. Older adults with a higher PR may also seek a return to equilibrium when facing difficult situations, which may further explain this link [48]. Further specific personality attributes linked to higher PR, such as seeking out meaning in life when facing adversity, may have encouraged older adults to seek out technologies [55]. For example, many church gatherings were web-based [18], and those seeking to find support from these would have to use technologies such as videoconferencing applications. Older adults with higher PR may have also used technology for “social exchange” having communicated with each other frequently to exchange opinions and information, especially on a subject such as the pandemic that was being covered and publicized heavily by media outlets.

EA also predicted greater TU after controlling for PR in our third hypothesis. Ross and Wu [95] suggested that education can provide students with the ability to communicate and analytical skills, gather and interpret information, and solve problems. Such skills increase older adults’ ability to control certain life events and outcomes and may contribute toward the ability to use technology. Our results are also consistent with prior research where education was found to increase the use of technologies and improve the quality of use, allowing a person to carry out higher-order tasks, potentially giving older adults a personal resource to overcome difficulties in using technology [73-75]. EA could also be a bridge that enables older adults to navigate and use technology more effectively. Technology represents advances in skills, knowledge, and abilities that change the manner in which a person performs tasks. It can be considered an acquired personal resource that ideally makes performing certain tasks easier, more efficient, safer, and perhaps pleasurable. Early education is linked to better computer skills and the ability to learn complex technologies more easily [72]. Therefore, our study cemented the importance of EA as a distal resource in later-life TU.

Our fourth hypothesis explored whether EA would moderate the impact of social isolation on loneliness. The direct effects of social isolation on loneliness in older adults are well established in the literature [6,8,11,12] and were confirmed in this study. We also saw that despite EA being associated with events in the past, it continued to play a significant role in moderating the impact of social isolation on loneliness. The moderating impact of EA on the effect of social isolation could be connected to the concept that older adults were persistent in maintaining contact with earlier studies, noting that people with higher EA tend to maintain high-quality social networks later in life [64,65]. Social relationships can be challenging and complex, and it is not unsurprising that people who have demonstrated the ability to be persistent with life goals and have developed accompanying emotional skills would be more likely to be able to maintain and manage social relationships, demonstrating how the pathway of EA as a distal resource impacts social isolation. EA may also have allowed people to articulate experiences, engage in social solidarity, seek appropriate social support, maintain a positive mindset, and apply previously learned skills [51,52]. Although the literature has provided some knowledge on explaining this relationship, this aspect of EA’s influence on social isolation requires further research.

The main principle of the DAM used in this study to structure our hypotheses highlights that resources shape risks and strengths to deal with difficulties [57] and indeed is an important consideration for successful aging [20,21]. We conjectured on this basis that EA could represent a type of distal resource that could dictate how susceptible older adults may be to loneliness because of its impact on proximal resources that were having a positive impact. To consider this, we examined whether EA would mediate the relationships between PR and loneliness and TU and loneliness. We found partial mediation by EA between TU and loneliness and complete mediation by EA between PR and loneliness. PR is known to be dependent upon high psychosocial functioning linked to EA [68], which allows older adults to maintain a positive disposition and achieve positive outcomes, despite the adversity of their circumstances. The strong link between higher cognitive function and EA later in life, which is essential to PR [71], may also explain the mediation effect. However, it should be noted that the unique circumstances of the COVID-19 pandemic may have amplified the impact of distal influences, as the immediate impact of the pandemic was overpowering, and other resources may have become diminished. Mirowsky and Ross [61] argued that the beneficial effect of a certain resource on another is greater when there is a situational context with resource limitation. They recognized that those with the fewest resources became more dependent on distal resources such as EA than those with more resources. Therefore, during the pandemic, when older adults’ resources were severely limited owing to social distancing and other adverse effects associated with the pandemic, EA may have played the role of a compensatory leveling resource, thereby playing a mediating role in PR in the absence of other resources. Thus, we observed the complete mediation impact of EA on PR during the pandemic, which was a notable result. Older adults with lower EA could be provided with further training and support, thereby benefiting them with the same impact as distal EA.

The mediation impact of EA observed between TU and loneliness was partly in line with our expectations. The ability of older adults to use technology can be challenging and often difficult to navigate, as the technological landscape is ever-evolving, with needs being placed on older adults to learn new skills at all times. For older adults, EA may have helped with navigation and understanding of the information they had access to through technological means or given them further abilities to communicate and engage, mediating the positive impact of technology on loneliness, in line with the DAM framework. However, we only saw a partial mediation of TU between EA and loneliness. With the lack of qualitative data, we can only conjecture about the possibilities for this result. During the pandemic, older adults may have found technology to have both a positive impact and a negative impact, depending on the type of information they encountered. There was an immense amount of media coverage of the pandemic on social media, with potential health threats to older people being advertised, as well as discussions and interactions that may have been both positive and negative on the topic. Therefore, it is perhaps unsurprising that the mediation impact of TU between EA and loneliness was only partial. However, more light could be shed on this through a structured qualitative study that examines TU and older adults’ perceptions of the information encountered and perceptions.

The indirect and direct effects of EA as a distal resource during the pandemic should be noted as a worthy outcome of our study and advance the knowledge associated with concepts behind successful aging. Previous research has focused on the influence of negative distal experiences such as family adversity and early traumatic events on developmental outcomes in older adults. However, EA in our study presented itself as a positive distal resource for older adults during the pandemic. EA is assumed to support increased mobility and opportunity (higher socioeconomic status) [96]; however, our study has shed new light on EA’s influence of EA on other proximal resources. Our findings support both the direct and indirect benefits of past EA on loneliness among older adults during the pandemic.

### Limitations

This study has several limitations. First, the sampling could have been predisposed to participants with access to or literacy in digital resources and potentially more socially connected individuals, at least virtually, owing to the recruitment process, which was largely coordinated on the web during the pandemic. These participants may have experienced less loneliness and may generally belong to a group with high EA. Second, a cross-sectional design cannot establish causality [97]. The absence of precise measures of PR, TU, loneliness, and social isolation from the period before the outbreak of the COVID-19 crisis for the same people precludes a comparison of the obtained data with prepandemic measures, which would also be useful in completing the picture.

The participants seemed to be representative of the age and gender distribution of the English population. Finally, one of the greatest weaknesses of the study was the lack of any measures of gross or adjusted household income or socioeconomic status based on occupational level (or previous occupational level for those who had retired), which could be linked to higher EA and potentially to higher availability and familiarity with technology. Higher household income would allow for affordability to access technologies and the internet and could add to the explanation of the impact of EA, leaving room for different interpretations and needs to be studied further.

### Conclusions

Despite several limitations, our study has practical value for professionals. Gerontologists, counselors, education providers, and aging service providers can use the findings of this study to determine those who may be more vulnerable to adverse circumstances, such as those created by the pandemic. EA in early life could have continued to provide insulating benefits against loneliness when faced with an adverse set of circumstances related to the pandemic. Lower EA and older age can be both considered factors influencing the “digital divide,” and this study demonstrates the importance of early-life education on later-life TU, suggesting that greater EA may help with technology adoption leading to the reduction of age-related gaps in the use of technologies. This, combined with EA’s relationship with PR, demonstrates that early-life investment in education helps older adults keep up with the increasing speed of technological developments, reducing the risk of digital exclusion, and increasing social participation through digital means.

Finally, one recommendation from this study is that when considering technology interventions for older adults, the study design should consider the EA of participants, as those with lower levels of EA may require more support and training to fully use the benefits of the impact of technology on loneliness. When EA is low, participants could benefit from additional later-life training and knowledge exchange that may have a similar impact.

### Suggestions for Future Research and Policy Implications

This study provides support for EA as an important resource for older adults. Our findings highlight the importance of EA in the early life course in influencing current resources. Education can help improve the older adult population’s health and well-being, including their ability to deal with life course or pandemic-linked social isolation and loneliness. Our study has provided support for ensuring that EA is not forgotten as an important resource for older adults in times of stress and isolation. EA also plays an important role in combating loneliness and social isolation and older adult use of technology. The literature on later-life cognitive decline strongly suggests that mid- and later-life learning and occupational complexity can act in place of distal EA [98]; therefore, there is a possibility of continued learning as a potential solution to the absence of distal EA, requiring further research.

Our contribution to research related to successful aging included how distal (education) and proximal resources influence the present-day experience of loneliness in older adults, and it can serve as a catalyst in the investigation of the impact of EA on other resources (eg, coping strategies, perceived optimism, perceived control, stress, anxiety, and depression) in older adults. Researchers should further understand the additional impact of distal educational experiences, particularly the level of complexity of education in older adults. They can continue to test and model the mechanisms by which EA may influence psychological well-being and loneliness in older adults. Longitudinal changes in loneliness relative to aging and lifelong and later-life learning experiences may provide a more accurate picture of the results of this study. Larger sample sizes with additional parameters such as gender, household income, and ethnicity will help form a richer picture.

This study adds to the body of research calling for policymakers to focus on education in the early stages of the life course to prevent social inequalities, loneliness, and isolation and encourage EA trajectories. It is essential for policy makers to include EA as a vital component in planning for pandemics in the future and to insulate older adults who are vulnerable to their consequences. Further interdisciplinary research into how EA impacts other current resources of older adults and how it can play a role in balancing health disparities is required, as well as research into compensating proximal factors such as continued learning later in life, which need to be explored.

## Abbreviations

DAM: developmental adaptation model
DCT: digital communication technology
EA: educational attainment
PR: psychological resilience
STROBE: Strengthening the Reporting of Observational studies in Epidemiology
TU: technology use
